# Recreational Cannabis Use During Human Pregnancy: Its Effects on the Placenta and Endocannabinoid System

**DOI:** 10.3390/ijms27031398

**Published:** 2026-01-30

**Authors:** Madhavi S. Harhangi, Lisa Höfert, A. H. Jan Danser, Hilmar H. Bijma, Sinno H. P. Simons, Irwin K. M. Reiss, Sven Baumann, Michelle Broekhuizen

**Affiliations:** 1 Department of Neonatal and Pediatric Intensive Care, Division of Neonatology, Erasmus MC University Medical Center, 3015 GD Rotterdam, The Netherlands; s.simons@erasmusmc.nl (S.H.P.S.); i.reiss@erasmusmc.nl (I.K.M.R.); m.broekhuizen@erasmusmc.nl (M.B.); 2 Department of Internal Medicine, Division of Pharmacology and Vascular Medicine, Erasmus MC University Medical Center, 3015 GD Rotterdam, The Netherlands; 3 Department of Obstetrics and Gynaecology, Division of Obstetrics and Fetal Medicine, Erasmus MC University Medical Center, 3015 GD Rotterdam, The Netherlands; h.bijma@erasmusmc.nl; 4 Institute of Legal Medicine, Medical Faculty, University of Leipzig, 04109 Leipzig, Germany; lisa.hoefert@medizin.uni-leipzig.de (L.H.); sven.baumann@medizin.uni-leipzig.de (S.B.); 5 Division of Neonatology and Pediatric Intensive Care, University Children’s Hospital Eppendorf, 20246 Hamburg, Germany

**Keywords:** cannabis, Δ^9^-tetrahydrocannabinol, cannabidiol, pregnancy, placental function, endocannabinoid system, fetal development, maternal health

## Abstract

The use of cannabis during pregnancy is increasing, in line with its growing societal acceptance and legalization. Cannabis use mainly concerns its active components Δ^9^-tetrahydrocannabinol (THC) and cannabidiol (CBD). While cannabis has therapeutic effects on pain, nausea, and vomiting, its impact on fetal development remains a significant public health concern. Given the existence of a local endocannabinoid system (ECS) in the placenta, with proven effects on placental development and blood flow, it is likely that THC and CBD exert effects via interference with the placental ECS. This review summarizes how cannabis use affects the placental ECS and describes the consequences of such use on placental function and fetal development. It starts with discussing the placental ECS, the effects of THC and CBD on placental function, and the pharmacokinetics of cannabinoids during pregnancy. It then describes the effects of both paternal and maternal cannabis use and provides epidemiological data linking placental insufficiency, impaired fetal growth, and preeclampsia to cannabis use. It also raises awareness for the possibility that cannabis use, by altering DNA methylation, might result in transgenerational effects. It is concluded that current evidence supports abstaining from cannabis use during preconception, pregnancy, and lactation to optimize maternal, fetal, and intergenerational health outcomes.

## 1. Introduction

Cannabis has a long history of medical use, including the treatment of dysmenorrhea and pregnancy-related nausea [1], and its use during pregnancy is controversial. Nowadays, the FDA has approved several cannabis-containing products like Marinol^®^ to stimulate appetite, Sativex^®^ for pain relief, and both Syndros^®^ and Cesamet^®^ for nausea and vomiting [2]. Indeed, cannabis seems to be effective for the treatment of pregnancy vomiting and morning sickness [3]. Yet its fetal and placental health risks should not be ignored [4,5], and thus with regard to the aforementioned drugs, it is often stated that they should be avoided during pregnancy. Remarkably, despite the latter, cannabis use among pregnant woman is currently increasing [6,7,8,9]. The COVID-19 pandemic might have contributed to this [10], together with cannabis legalization and the increased societal acceptance of cannabis use [11]. Therefore, it is important to summarize what is known and to identify where important knowledge gaps remain.

The active components of *Cannabis sativa* are referred to as (phyto)cannabinoids [12,13]. At least 144 have been identified [14], with Δ^9^-tetrahydrocannabinol (THC) and cannabidiol (CBD) being the most studied ones [12]. Importantly, the human body possesses its own endocannabinoid system (ECS), and cannabinoids are likely to exert the same effects as endocannabinoids, e.g., affecting inflammation, appetite, mood, and stress [15,16]. Moreover, endocannabinoids and plant-derived cannabinoids share structural similarities, like lipophilicity, hydrophilic heads, and long fatty acid chains [17,18]. In this review, we will refer to phytocannabinoids as “cannabinoids” and endogenous cannabinoids as “endocannabinoids.”

A local, endogenous ECS is present in the placenta [19,20]. The placenta is a vital organ that develops during pregnancy, facilitating the exchange of nutrients, gases, and waste products between the mother and fetus, while also producing hormones essential for maintaining pregnancy [21]. It serves as a barrier and regulator of substances that pass between maternal and fetal blood, playing a critical role in fetal development. The placental ECS is believed to regulate cell growth, differentiation, and immune responses [19]. To what degree maternal endocannabinoids affect or cross the placenta is currently unknown. Yet, certain cannabinoids have been reported to cross the placenta [22,23].

This review aims to explore the effects of maternal cannabis use on endocannabinoid/cannabinoid signaling of the placenta during pregnancy in order to understand why cannabis use would affect placental function and fetal development. After introducing the placental ECS, the effects of CBD and THC on placental functional processes are discussed. Next, we focus on their pharmacokinetics and summarize the clinical outcomes of maternal and paternal cannabis use, also considering the long-term effects of such use, i.e., after birth. The review ends with an overall conclusion and future outlook.

## 2. Material and Methods

A literature search was performed in Pubmed with the following search terms: (“cannabis”[MeSH Terms] OR “cannabis”[All Fields] OR “cannabi”[All Fields] OR “cannabis s”[All Fields]) AND (“placenta”[MeSH Terms] OR “placenta”[All Fields] OR “placentas”[All Fields] OR “placenta s”[All Fields] OR “placentae”[All Fields]). All papers were sorted based on the following categories: functional and cellular effects of cannabis use on the placenta, metabolism and transport, pharmacokinetics, and epidemiology.

## 3. The Endocannabinoid System in the Placenta

**Figure 1:**
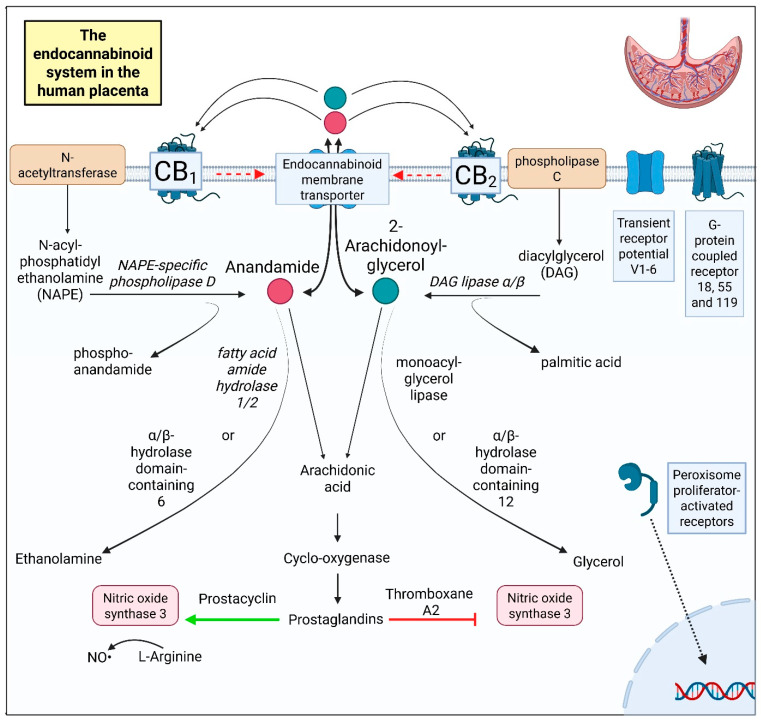
A schematic overview of the ECS in the human placenta. Figure was made with Biorender.com. Abbreviations: CB1, cannabinoid receptor 1; CB2, cannabinoid receptor 2; NO, nitric oxide.

A schematic overview of the ECS is shown in Figure 1. The placental ECS is not unique, as an independent ECS has also been described in the brain and liver [24]. Endocannabinoids are lipid-signaling molecules produced from membrane phospholipids [19,25,26,27]. *N*-acetyltransferase and phospholipase C convert these membrane phospholipids into *N*-acyl phosphatidylethanolamine and diacylglycerol, respectively. In turn, these products are converted into the two major endocannabinoids, anandamide (AEA) and 2-arachidonoylglycerol (2-AG), by the enzymes *N*-acyl phosphatidylethanolamine (NAPE)-specific phospholipase D and 1,2-diacylglycerol (DAG) lipase α/β. AEA is subsequently hydrolyzed into ethanolamine by fatty acid amide hydrolase (FAAH) 1 or 2, with FAAH1 displaying greater hydrolytic activity than FAAH2 [28,29]. 2-AG is metabolized by monoacylglycerol lipase (MAGL) [19]. Minor routes of AEA and 2-AG hydrolyzation involve α/β-hydrolase-6 and -12. The byproduct of the hydrolyzation of these endocannabinoids is arachidonic acid, which can be metabolized by cyclooxygenase into prostaglandins [30,31]. Some prostaglandins are capable of inducing nitric oxide production in endothelial cells, which can contribute to vasodilation, while others can also inhibit nitric oxide synthase 3, leading to vasoconstriction [32]. Other endocannabinoids include the N-acylethanolamines palmitoyl ethanolamine and oleoyl ethanolamine, but their biological role is incompletely understood [33].

Endocannabinoids can be transferred in and out of the cell via the endocannabinoid membrane transporter (Figure 1). Extracellular endocannabinoids interact with cannabinoid receptors 1 and 2 (CB1 and CB2) on the cell membrane. Both receptors occur in the human placenta [34,35], where they are expressed in cytotrophoblasts, syncytiotrophoblasts, and extravillous trophoblasts, as well as in endothelial cells [26,36]. They contribute to trophoblast differentiation [37], migration [38], and invasion [39] but are also involved in mitochondrial function [40], apoptosis [37], and angiogenesis [41]. Both CB1 and CB2 signal through the extracellular signal-regulated kinase pathway [42,43] and vascular endothelial growth factor (VEGF) [41].

The transient receptor potential cation channel subfamily V (TRPV) can also be affected by endocannabinoids [44]. All six (TRPV1-6) channels are expressed in the placenta [35]. TRPV1 is expressed in cytotrophoblasts and syncytiotrophoblasts and induces caspase 3 and 7 activation [45], thereby affecting apoptosis [46] and trophoblast viability [45]. Moreover, TRVP1 co-localizes with CB1, thus potentially influencing its downstream effects [44]. The role of TRPV2 and TRPV3 in the human placenta is not yet fully understood [47], although TRVP2 might play a role in placental development [48]. TRPV4 is found in both trophoblasts and endothelial cells, and it is important for vascular function [47,49]. TRPV5 is mainly found in Hofbauer cells, while TRPV6 occurs in trophoblasts and endothelial cells [47]. Both TRPV5 and TRPV6 are involved in transcellular calcium transport [50].

Three orphan receptors are also considered part of the placental ECS [51]. The first is G-protein coupled receptor 55, which is now also known as cannabinoid receptor 3 [52]. Although it lacks the typical cannabinoid receptor binding pocket [53], it binds cannabinoids like AEA [54,55] and was reported to play a role in endothelial cell migration in the placenta [53]. The second cannabinoid-related receptor is G-protein coupled receptor 18 [56]. It is considered a cannabinoid-related receptor. This receptor is expressed in decidual cells, vascular smooth muscle cells, and extravillous trophoblasts and has an anti-inflammatory role [57]. The third orphan receptor is G-protein coupled receptor 119 [51]. Remarkably, it could only be detected at the protein and not the mRNA level in the placenta [58,59].

Nuclear receptor peroxisome proliferator-activated receptor gamma (PPARγ) might also associate with the ECS. It can bind cannabinoids and occurs in cyto- and syncytiotrophoblasts, affecting their differentiation and invasion [60,61,62]. Nevertheless, it is not considered a cannabinoid receptor [35].

Finally, opioid receptor μ may bind endocannabinoids and/or interact with CB1 [63]. It has been demonstrated in placental tissue, although its specific location is not known [64].

### Endocannabinoid Imbalance

The ECS plays a key physiological role in early pregnancy [19]. Because these processes depend on tightly controlled receptor signaling, precise regulation of the ECS is required for successful implantation and proper placental development [19]. Elevated systemic AEA levels inhibit blastocyst attachment and trophoblast differentiation [65,66]. AEA plasma levels are higher in women with miscarriages compared to women with live births [66]. Dysregulated endocannabinoid metabolism occurs in preeclampsia and fetal growth restriction, where elevated CB1 expression and altered FAAH1 activity might contribute to defective trophoblast invasion and insufficient uterine vascular remodeling [67,68]. The elevated FAAH2 levels observed in preeclampsia [69] might underlie the augmented inflammatory responses in this disorder [70]. In pregnant woman where the ECS balance is critical, cannabis use can lead to dysregulation of the maternal immune system [71].

## 4. Functional and Cellular Effects of Cannabis Use on the Placenta

The placenta contains many different cell types that interact with each other [72]. On the maternal side, the uterine compartment contains maternal endometrial and decidual cells as well as fetal extravillous trophoblasts that invade the uterine lining at the beginning of pregnancy and aid in the remodeling of spiral arteries, which fill the intervillous space with maternal blood from the second trimester onwards. The intervillous space contains the fetoplacental villous trees, which are lined by a syncytiotrophoblast layer bathing in maternal blood. Beneath the syncytiotrophoblast layer is a cytotrophoblast layer, and the villous core contains mesenchymal cells, which include the fetal endothelial cells that form the fetoplacental vasculature.

### 4.1. Placental Endocrine Effects and Effects on the Placental ECS

To the best of our knowledge, there is no strong association between cannabinoid use and changes in endocrine function of the placenta. In women who smoked cannabis during pregnancy, the plasma concentrations of the placental hormones human chorionic gonadotropin, pregnancy-specific beta-1-glycoprotein, human placental lactogen, progesterone, 17-hydroxyprogesterone, estradiol, and estriol were unaltered compared to matched controls throughout all trimesters of pregnancy [73]. However, there was a wide range in the frequency of cannabis use in the group of women who used cannabis during pregnancy, and a potential dose-dependent effect can therefore not be excluded. At high concentrations in vitro (>20 µmol/L), THC was reported to impact placental estrogen signaling, which plays important roles in placentation and placental development [74]. In both villous explants and BeWo cells (a human cell line derived from a placental choriocarcinoma), THC resulted in enhanced estrogen signaling through upregulated expression of one of the rate-limiting enzymes in estrogen production, cytochrome P450 aromatase, in an estrogen receptor α- and CB1-dependent manner [74]. Yet, in endometrial stromal cells, THC had no effects on estrogen signaling, while CBD was reported to inhibit endothelial stromal cell differentiation by attenuating the physiological upregulation of estrogen signaling [75]. These opposing effects of THC and CBD suggest cell type-specific mechanisms that have yet to be uncovered. Altogether, cannabis constituents may affect placental endocrine functions, particularly estrogen signaling, and these effects may explain infertility and pregnancy problems in women who use cannabis during pregnancy.

With regard to the expression levels of ECS components (including CB1 and CB2), only NAPE-specific phospholipase D was upregulated in placental villous biopsies from mothers who used cannabis during pregnancy compared to healthy placentas [20]. Remarkably, despite the unaltered CB1 and CB2 expression, AEA and 2-AG predominantly appeared to signal through non-CB receptors [20].

### 4.2. Trophoblast Migration and Implantation

Initial placental development depends on tight regulation of trophoblast proliferation, differentiation, migration, and invasion [76]. These processes are known to involve endocannabinoid components and are thus likely to be affected by exogenous cannabis constituents. In women who smoked cannabis during pregnancy—and did not use tobacco or alcohol—the placentas presented with an increased number of syncytiotrophoblast knots and fibrin exudation in the villous stroma [38]. These pathological changes may contribute to an impaired placental transfer of oxygen, nutrients, and waste products, potentially hampering fetal growth.

In vitro studies with cell lines indicate that THC causes impaired trophoblast development. Several studies have reported that THC can inhibit proliferation, impair mitochondrial respiration, and induce oxidative stress in cytotrophoblasts (primary and BeWo cells) at concentrations of 20 µmol/L and higher [37,77,78]. Importantly, THC was also shown to attenuate syncytialization [37], the fusion and morphological differentiation of cytotrophoblasts into syncytiotrophoblasts, as well as reduce the capacity of trophoblasts to migrate and invade [38,79,80], all of which are crucial events in placental development. THC may, however, have biphasic effects in trophoblasts [81]. One study reported enhanced 3-(4,5-dimethylthiazol-2-yl)-2,5-diphenyltetrazolium bromide metabolism (an indicator of cell viability), reduced reactive oxygen species production, and increased adenosine triphosphate production in primary trophoblasts at THC concentrations at or below 20 µmol/L [37]. In mice, THC was also shown to impact trophoblast stem cell differentiation in a CB1-dependent manner [82].

The effects of CBD seem to mimic those of THC. Indeed, it also decreases cell viability, proliferation, and mitochondrial respiration, associated with disruption of the mitochondrial membrane potential, increased oxidative stress and autophagy at concentrations at or above 5 µmol/L [83,84]. Moreover, CBD (at concentrations > 2 µmol/L) impaired differentiation and migration of cytotrophoblast- and syncytiotrophoblast-like cell lines, i.e., BeWo and HTR-8/SVneo [80,83,84].

It is important to note that the concentrations of CBD and THC applied in these studies are significantly higher compared to the levels measured in persons who smoke cannabis. THC was reported to reach peak serum concentrations > 100 ng/mL (320 nmol/L) [85,86,87], while CBD concentrations appear to be lower (>2 ng/mL; 6 nmol/L) [87]. It should, however, be noted that both chemicals are highly lipophilic, and thus it is possible that they accumulate in fat tissue at higher concentrations. These data indicate that cannabis abuse may impact trophoblasts’ differentiation and implantation.

It is not only the trophoblasts that determine the success of implantation of an embryo. Endometrial cells undergo a process called decidualization, which is important to create a receptive uterine environment [88]. This process can be affected by cannabinoids. THC and CBD are toxic to decidualizing-immortalized human endometrial stromal cells at concentrations > 20 µmol/L [39]. Even at a non-toxic and more clinically relevant concentration of 0.5 µmol/L [85,86], THC and CBD inhibited decidualization, delayed the attachment of trophoblasts spheroids to decidualized endometrial cells, and reduced the invasive capacity of trophoblasts into the decidualized endometrial cells [39]. One other study did not report any effects of THC on decidualization of immortalized endometrial cells, potentially due to the application of lower concentrations (<10 µmol/L) [75]. They did, however, find that CBD attenuated endometrial cell differentiation through inhibition of the physiological increase in estradiol production in differentiating endometrial cells [75].

**Figure 2:**
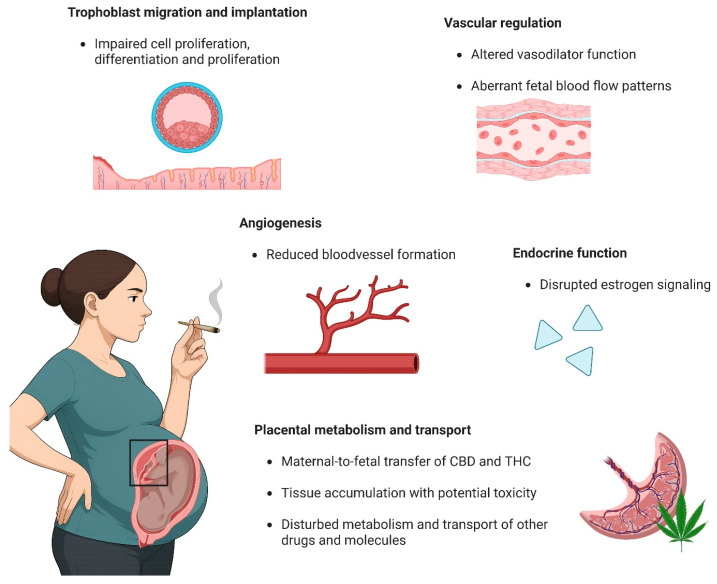
An overview of the functional and cellular effects of cannabis use on the placenta. Figure was made with Biorender.com. Abbreviations: CBD, cannabidiol; THC, Δ^9^-tetrahydrocannabinol.

Altogether, these data suggest that THC and CBD can affect reproductive capacity by impairing cell proliferation, differentiation, and migration of trophoblasts as well as endometrial cells. An overview of all functional and cellular effects of cannabis use on the placenta can be found in Figure 2.

### 4.3. Angiogenesis

Cannabis use during pregnancy may cause impaired angiogenesis in the placenta. Pregnant women who smoked cannabis presented with a more narrow placental vascular network [89]. Similar features were visible in pregnant mice that were injected with 5 mg/kg THC throughout pregnancy [89]. Yet, at a lower dose (3 mg/kg), THC mainly decreased the ratio between the fetal and maternal blood area in an increased placental labyrinth layer and reduced the expression of trophoblast labyrinth progenitor marker *Epcam* [90]. At the same dose, CBD did not cause changes as prominently as THC, although it still resulted in a relatively lower fetal blood space perimeter and reduced expression of the endothelial marker CD31 [91]. These changes were also associated with downregulation of angiogenesis and blood vessel formation-related processes, including responses to growth factors, NO signaling, and mitogen-activated protein kinase activity [91]. In vitro, THC reduced cell viability, impaired migratory capacity, and reduced tube length formation of human umbilical vein endothelial cells, potentially by inhibiting of the RhoA pathway [89]. Similarly, CBD inhibited endothelial cell proliferation, migration, and VEGF-dependent capillary sprouting [92].

In summary, these data suggest that both THC and CBD may directly impair blood vessel formation and angiogenesis, leading to a reduced fetal blood surface area in the placenta and impairing fetal development (Figure 2).

### 4.4. Vascular Regulation

Both exogenous and endogenous cannabinoids can affect vascular tone. It is known that cannabis—in particular THC—causes an acute increase in heart rate, blood pressure, and arterial stiffness in humans [93,94]. A recent study also found that THC, but not CBD, increased post-exercise pulse pressure and left ventricular contraction duration [95]. These data indicate that THC has significant cardiovascular effects, which may impact pregnant women and their offspring. Although experimental studies in pregnant women are unethical, observational studies found a higher uterine pulsatility index in women who used cannabis throughout pregnancy compared to those who did not or smoked tobacco [96,97]. Additionally, continuous cannabis use was associated with fetal blood flow changes including an elevated umbilical artery pulsatility index (Umb Art PI), a decreased middle cerebral artery pulsatility index (MCA PI), and a consequently lower cerebral placental ratio (calculated as MCA PI/Umb Art PI), accompanied by a reduced fetal aorta inner diameter and lower pulmonary peak systolic velocity [96,97,98]. It should, however, be noted that these fetal aberrancies were also present in pregnant women who smoked tobacco during pregnancy [96,97], and thus it cannot be excluded that these effects were actually mediated by tobacco. In a non-human primate model, prenatal THC administration was not associated with changes in umbilical and uterine Doppler measurement. However, this study did observe reduced placental perfusion and fetal oxygen availability as measured by MRI, accompanied by a reduced amniotic fluid volume, increased placental micro-infarctions, and transcriptional changes in the placenta associated with alterations in cytokine binding, regulation of cell migration, cell-substrate adhesion, angiogenesis, and vascular development [99]. These data support the idea that THC can induce a degree of placental insufficiency.

These vascular changes may directly result from an effect of exogenous cannabinoids via the vasodilator properties of the endocannabinoid system. Krzyżewska et al. [29] summarized that AEA and 2-AG can alter vascular tone via endothelial nitric oxide synthase, CB1, calcium-dependent potassium channels, and TRPV channels or indirectly when converted into prostaglandin I2 and thromboxane A2. Recently, we identified that AEA and 2-AG can also dilate the fetoplacental blood vessels of healthy pregnancies [20]. In cannabis users, however, the responses to 2-AG was greatly diminished, and dilation to AEA was even entirely absent [20]. These data suggest that cannabis use during pregnancy significantly impacts vascular regulation, although a full understanding of the separate effects of all cannabis components is still lacking.

THC has been reported to have vasodilatory effects [100,101,102,103]. Yet, the effects of cannabinoids appear to differ between large and small arteries and potentially between in vivo and in vitro conditions. O’Sullivan et al. [104] showed that vasodilation to THC was PPARγ- and endothelial-dependent but did not involve CB1 in the rat aorta and superior mesenteric artery. Besides direct effects, THC was also reported to affect responses to other agonists in an opposite manner in large conduit and small resistance arteries. Indeed, while THC amplified constriction to methoxamine and inhibited vasodilation to acetylcholine in resistance arteries, in conduit arteries, it attenuated constriction and had no effect on vasodilation [105]. Despite the THC-induced dilation observed ex vivo, THC caused CB1-mediated renal and mesenteric vasoconstriction in conscious rats [106]. Furthermore, although THC-induced vasodilation was enhanced in dissected arteries from N-nitro-L-arginine methyl ester-treated rats compared with controls, these effects were absent in vivo [106]. These discrepancies likely arise from interactions between cannabinoids and the systemic nervous system, which is missing in ex vivo preparations but which should be considered when performing such studies.

In addition, CBD was shown to dilate blood vessels, relying on mechanisms that greatly differed between tissues. Similarly to THC, CBD dilated the rat aorta in a PPARγ-dependent manner, although in this case, the response was not dependent on the endothelium but seemed to involve Ca^2+^ channel inhibition [107]. In human mesenteric arteries, CBD induced dilation via CB1, which was endothelium- and nitric oxide-dependent [108]. CBD also dilated larger human pulmonary arteries endothelium-dependently, although this was mediated through TRPV1 and not CB1 [109].

The data summarized above provide evidence that both THC and CBD can dilate arteries (Figure 2). However, the exact effects of these substances on the placental vasculature remain to be uncovered, as their mechanisms seem to differ largely depending on the tissue origin or the size of the blood vessel or may even change with disease, as indicated by our recent data in preeclamptic women [20].

## 5. Placental Metabolism and Transport

Placental metabolism and transport are important processes that affect the exposure of the fetus to many biological and chemical compounds during pregnancy. Besides the potential transfer of cannabinoids to the fetal circulation when the mother uses cannabis during pregnancy, cannabinoids may also impact fetal development by altering placental metabolism and transport functions, potentially changing the fetal exposure to many other substances.

It has been demonstrated that when administered during pregnancy, THC transfers from maternal to fetal plasma and accumulates in fetal tissues and amniotic fluid [110,111,112]. In late-term non-human primates, THC crossed to the fetal circulation, reaching equal concentrations to the mother’s 3 h after administration, and accumulated in all fetal tissues—including the placenta—and in most organs at higher concentrations than plasma levels [112]. Interestingly, a human study of women who smoked cannabis in the last trimester demonstrated that cord blood levels of THC were up to six times lower than maternal levels [113]. In an open-circuit human placenta perfusion setup, Kumar et al. [22] found that the fetal-to-maternal clearance of THC was higher than the maternal-to-fetal clearance, suggesting that the THC concentrations would be lower in the fetal versus the maternal circulation in vivo. Importantly, their data also suggest that the placenta does not metabolize THC into either 11-hydroxy-THC (11-OH-THC) or 11-nor-9-carboxy-THC (THC-COOH), although both of these metabolites seem to passively transfer between the maternal and fetal circulations [22]. A preferential efflux of THC from the fetal into the maternal circulation was confirmed in a follow-up study, where the same authors predicted that THC would have a fetal-maternal plasma ratio 0.35 ± 0.13 in the third trimester by applying a maternal-fetal physiologically based pharmacokinetic model [114]. It thus appears that the placenta contains an efflux mechanism that prevents the fetus from being exposed to the same THC levels as the mother. The responsible efflux transporters remain to be determined, but studies have excluded a potential role for P-glycoprotein (P-gp; ABCB1), breast cancer resistance protein (BCRP; ABCG2), organic anion-transporting polypeptide 2B1, organic cation transporter 3, or organic anion transporter 4 [113,115,116]. THC’s metabolites, 11-OH-THC and THC-COOH, however, passively diffused across the placenta [113]. Moreover, it should be noted that THC and THC-COOH appear to be organic cation transporter 1 substrates and therefore may cause organic cation transporter 1-related drug interactions [115]. THC was also shown to noncompetitively inhibit amino acid uptake in term placentas, potentially hampering growth of the placenta and fetus [117].

The transfer of CBD has been studied in an ex vivo human placenta perfusion setup. Berman et al. [23] found that after 180 min dual closed-loop perfusion, the fetal levels corresponded with approximately 20% of the maternal levels, and they reported significant accumulation of CBD in placental tissue. Unfortunately, this study reports no data on the transfer of CBD before 180 min, and thus it cannot be concluded whether a steady state had been reached.

In the same study, Berman et al. found no effect of CBD exposure on the expression of many transporters after 180 min perfusion [23]. This is in contrast with earlier findings in vitro with BeWo cells where CBD—applied at >3 fold higher concentrations—reduced the expression of efflux transporter and anti-apoptotic P-gp, while it increased the expression of the anti-apoptotic BCRP [118]. In another ex vivo human placenta perfusion study, CBD reversed the preferential transfer of glyburide towards the maternal circulation, into a preferential transfer towards the fetal circulation, which would result in a significantly higher fetal glyburide exposure [119]. Based on this, it was proposed that cannabis use during pregnancy may affect the transplacental transfer of (therapeutic) drugs and other molecules that are P-gp or BCRP substrates, making the safety of some drugs that are considered safe during pregnancy questionable. Besides transfer mechanisms, CBD may also impact placental metabolic processes. In vitro administration of CBD to human term placental explants resulted in disrupted tryptophan metabolism into either kynurenine or serotonin [120], both of which appear to require a tight regulation for healthy pregnancy outcomes [121,122].

In conclusion, both THC and CBD concentrations in the fetal circulation are predicted to reach 20–30% of the maternal plasma circulations. This relatively low maternal-to-fetal transfer cannot be taken as evidence for their safety. Their apparent accumulation in the placental and other fetal tissues [114] points towards potential toxic effects, and their indirect effects on other transport mechanisms and metabolism warrant further research.

## 6. Cannabis and Cannabinoid Pharmacokinetics in Pregnancy

The pharmacokinetics and metabolism of CBD and THC in non-pregnant humans are well studied [14]. Other cannabinoids include cannabichromene, cannabidivarin, and cannabigerol [123], although far fewer data are available regarding their disposition. Given that CBD and THC account for the majority of recreational and therapeutic exposure, this section focuses on the pharmacokinetics of these two compounds in pregnancy.

**Figure 3:**
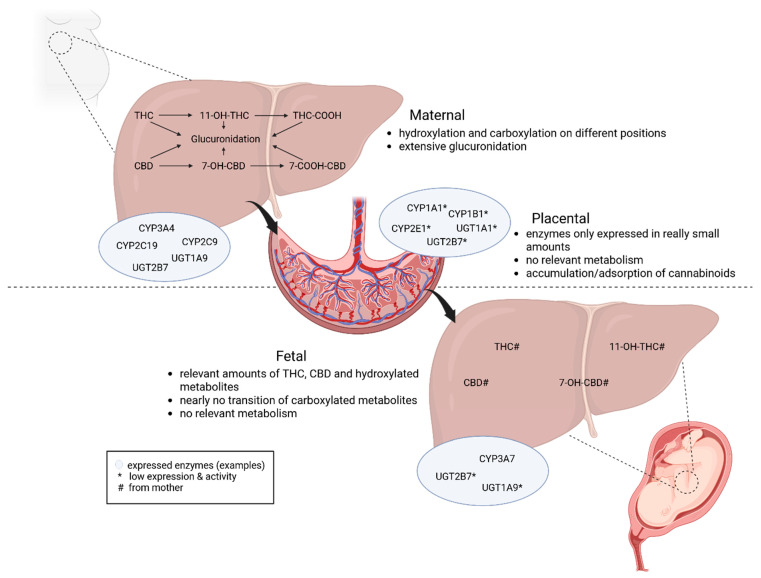
An overview of the pharmacokinetics of CBD (cannabidiol) and THC (Δ^9^-tetrahydrocannabinol) during pregnancy, with indication of enzymes involved in the maternal liver, placenta, and fetal liver. Figure was made with Biorender.com. CYP: cytochrome P450; UGT: Uridine 5′-diphospho-glucuronosyltransferase.

Pregnancy and lactation introduce substantial physiological changes that can modify all pharmacokinetic components, including the absorption, distribution, metabolism, and elimination profile of cannabinoids [124,125]. To contextualize mechanistic and clinical findings, this section summarizes current knowledge on the maternal, placental, and fetal pharmacokinetics of THC and CBD. An overview of cannabinoid metabolism and involved enzymes in the maternal liver, fetal liver, and placenta during pregnancy can be found in Figure 3.

### 6.1. Maternal

The absorption of cannabinoids depends on the route of administration. After inhaled consumption, e.g., as a joint or using a vaporizer, THC is absorbed rapidly, resulting in high plasma peak concentrations after 3–10 min [126,127,128]. The bioavailability for this consumption method is around 10–35% [127,129]. In contrast, after oral consumption as so-called edibles, e.g., brownies or fruit gums, the bioavailability is only around one-third compared to inhaled consumption due to an extensive first-pass metabolism [130,131,132]. Additionally, the plasma peak concentrations are lower and reached only after around 1–3 h after oral consumption [126,127,129,132,133]. The way of consumption is not only important for the pharmacokinetics but might also impact the overall fetal toxicity due to different peak concentrations and metabolite patterns [132].

Most cannabinoids, like CBD and THC, are highly lipophilic, which leads to fast uptake into the central nervous system and large volumes of distribution in the body due to an extensive accumulation in fat tissue [125,134,135]. During pregnancy, plasma volume, total body volume, and fat mass are increased, resulting in an increased volume of distribution which might lead to decreased maternal cannabinoid plasma concentrations. However, the plasma protein binding to albumin might be decreased in pregnancy, which can result in an increased free fraction of the cannabinoids and their metabolites in pregnant women [136].

Regarding the metabolism of cannabinoids, both the cytochrome P450 (CYP) system and uridine 5′-diphospho(UDP)-glucuronosyltransferase (UGT) enzymes play a major role. THC and CBD are mainly oxidized by CYP2C9, CYP2C19, and CYP3A4, resulting in different hydroxy- and carboxy-metabolites [127,129]. The quantitatively most important ones are 11-OH-THC and THC-COOH for THC and 7-hydroxy-CBD (7-OH-CBD) and 7-carboxy-CBD (CBD-COOH) for CBD [137]. Afterwards, extensive conjugation via glucuronidation occurs at various positions of the phase 1 metabolites to promote the excretion of the substances via urine and feces [87,127,137]. During pregnancy, the activity of different enzymes can be either increased or decreased, which leads to possible changes in clearance kinetics and metabolism [124,125,138]. Recent research showed that CYP2C9 and CYP3A4 in particular are known for an induction by pregnancy hormones, which might lead to a different disposition of THC and 11-OH-THC as well as different pharmacodynamic effects [124]. Additionally, it is important to mention that cannabinoids, in particular CBD, are able to inhibit the catalytic activity of human CYP3A enzymes, which might impact the metabolism and toxicity of other substances [139].

Cannabinoids primarily undergo biliary and renal excretion. During pregnancy, the glomerular filtration rate is increased, which results in an enhanced renal elimination of cannabinoid conjugates [124,140]. A simulation study showed that the area under the curve of THC did not change during pregnancy, but the area under the curve of the psychoactive metabolite 11-OH-THC decreased by up to 41% [141]. However, the data on specific plasma half-lives and other pharmacokinetic parameters in pregnant women are limited.

### 6.2. Placental

Cannabinoids like CBD and THC are relatively small, lipophilic, and non-ionized compounds, which can therefore diffuse passively across membranes such as the placental syncytiotrophoblast. Placental permeability tends to increase in later stages of pregnancy, largely due to thinning of the trophoblast barrier and a reduction in the diffusion distance between maternal and fetal circulation [142,143].

The placenta expresses a limited amount of CYP and UGT enzymes [144,145]. Therefore, oxidative metabolism is most likely quantitatively negligible [145]. Overall, placental metabolism plays only a minor role, and the main metabolic burden remains maternal [144].

### 6.3. Fetal

As previously mentioned, CBD and THC can cross the placenta, which leads to measurable fetal plasma and tissue levels of these substances. Especially in cases of chronic maternal cannabis consumption, cannabinoids and their metabolites can accumulate in fetal tissues [23,114]. Due to their lipophilicity, the cannabinoids accumulate preferentially in lipid-rich organs such as the brain, adipose tissue, and liver [112,114,132]. It is also possible to detect cannabinoids in, e.g., umbilical cord blood, meconium, neonatal hair, and the placenta itself [23,114,132].

Various studies with different animal models have shown that after maternal exposure, the fetal cannabinoid blood levels are typically lower and the maxima reached later compared to the maternal levels [112,125,146,147,148]. Human data are very limited, presumably mainly due to ethical reasons, which makes it difficult to conduct controlled consumption studies.

Like the human liver, the fetal liver expresses CYP and UGT enzymes, albeit at low levels [149,150,151]. Furthermore, the enzyme profile in the fetal liver is different compared to that in the livers of newborns, children, and adults. The predominant CYP enzyme in the fetal liver is CYP3A7, while it is CYP3A4 in adults [150,152]. Additionally, UGT enzymes are hardly expressed in the fetal liver [153]. This indicates differences in the metabolic capacity, potentially leading to a slower oxidative metabolism and a prolonged persistence of cannabinoids in the fetal system.

In summary, various changes during pregnancy can affect the pharmacokinetics and metabolism of cannabinoids. Further research is needed to complete the existing data and to comprehensively assess the potential risks of cannabis consumption during pregnancy. Additionally, it is important to mention that current data only apply to frequently studied cannabinoids such as THC and CBD. Completely different circumstances may apply to semi-synthetic or synthetic cannabinoids.

## 7. Societal Impact of Cannabis Use

In the United States, 5.3% of pregnant women reported cannabis use in 2015–2019 National Survey on Drug Use and Health data [154]. European estimates range from 2.6 to 5.0% [155]. Cannabis use during pregnancy is associated with younger age, single status, use of cannabis by the partner of the pregnant woman, childhood trauma, lower education, and poverty [6,155,156,157]. Women report coping, symptom relief, and recreational motives, and as discussed, misconceptions about safety are common [158,159].

### 7.1. Preconception Paternal Cannabis Use

Paternal cannabis use preconception is common; approximately one in four men in a recent North American preconception cohort reported use [160]. Evidence for effects on semen quality is inconsistent: population-based data show no clear deficits in semen volume, concentration, motility, or total motile count, whereas clinic-based studies report adverse associations such as reduced morphology, lower ejaculate volume, and erectile dysfunction [161,162,163]. Overall, findings are heterogeneous and appear to depend on user characteristics, frequency, and study population.

Cannabis exposure has been linked to altered DNA methylation in sperm, though the clinical significance of these epigenetic modifications remains uncertain [164,165,166]. Some cohort data suggest an increased risk of miscarriage with paternal preconception cannabis use, although confounding by tobacco and other substances cannot be excluded [167].

### 7.2. Preconception Maternal Cannabis Use

Preconception cannabis use is reported in 5.6–11.3% of women [157,168]. Emerging evidence links use with endocrine and reproductive alterations, including decreased luteinizing and follicle-stimulating hormone levels and impaired ovulatory function [163]. Recent human in vivo and in vitro data show that cannabinoids can interfere with oocyte maturation and fertilization potential [169]. These findings align with reports of reduced fecundability and higher miscarriage risk among female users [167,170].

### 7.3. Cannabis Use During Pregnancy

Maternal cannabis use during pregnancy has been associated with hypertensive disorders, preeclampsia, and placental abruption in large contemporary cohorts [157]. Fetal and neonatal outcomes show lower birth weight, small-for-gestational-age status, and increased neonatal intensive care unit (NICU) admission [171,172]. A recent population-based study involving over 360,000 pregnancies confirmed these associations, reporting dose-dependent increases in the risk of low birth weight and small-for-gestational-age infants, modest elevation in preterm birth, and higher NICU admission rates among cannabis-exposed neonates [173]. Beyond immediate obstetric risks, intrauterine exposure to cannabinoids may influence fetal neurodevelopment and behavioral trajectories across the life course [132,172,174].

### 7.4. Neonatal Transition and Early Adaptation

Neurobehavioral findings are heterogeneous. Some studies using the Neonatal Behavioral Assessment Scale report transient increases in tremulousness, exaggerated startle, and lower autonomic stability, whereas others find no differences [175]. When present, these signs typically resolve within days and are distinct from opioid-type withdrawal. Lo et al. interpret such transient dysregulation as a short-term effect of abrupt cessation of fetal cannabinoid exposure at birth [172].

Epigenetic data further suggest altered DNA methylation at placental and fetal genes regulating vascular and trophoblastic function, supporting a possible molecular pathway linking prenatal exposure with neonatal adaptation differences [174].

### 7.5. Breastfeeding

THC and other cannabinoids are excreted into human breast milk at concentrations several-fold higher than in maternal plasma, owing to their lipophilicity and affinity for milk fat. THC has been detected in breast milk from 6 days up to more than 6 weeks after maternal use, with reported milk-to-plasma ratios around 6:1 and modeled elimination half-lives extending beyond 17 days [176,177,178].

Infants exposed via breastfeeding demonstrate measurable urinary THC metabolites and, in small longitudinal cohorts, possible transient differences in motor development or arousal regulation, although results are inconsistent and confounded by concurrent prenatal exposure [178,179]. These subtle neurobehavioral influences may arise from effects on CB1 and CB2, which are expressed in the neonatal brainstem and hypothalamic regions, governing suckling, thermoregulation, and sleep, providing biological plausibility for subtle neurobehavioral influences [180,181].

Given the persistence and accumulation of cannabinoids in breast milk, their potential effects on the developing brain, and the lack of a defined safe threshold, abstinence from cannabis during breastfeeding is strongly recommended [177,178].

### 7.6. Long-Term Effects

As mentioned before, the fetal liver shows minimal expression of THC-metabolizing cytochromes, limiting its clearance capacity in utero [182,183]. This immaturity, together with the lipophilicity and long half-life of cannabinoids, supports tissue accumulation and prolonged exposure during critical periods of brain development. Such exposure is likely to have chronic effects by disrupting endocannabinoid-regulated processes such as neuronal migration, synaptogenesis, and axonal pruning—core mechanisms in cortical and limbic circuit formation, which are essential for proper brain development [184].

Longitudinal epigenetic studies demonstrate persistent methylation differences at neurodevelopmental and placental genes among exposed offspring [164,174]. Although peripheral, these methylation changes suggest systemic developmental re-programming that may underlie later neurobehavioral effects.

### 7.7. Transgenerational Effects

During pregnancy, cannabinoids not only affect the developing fetus but also reach the fetus’s germ cells—the primordial oocytes or spermatogonia that will generate the next generation. These cells express CB1 and CB2, which regulate proliferation and maturation [185,186]. Experimental activation of these receptors alters the timing of meiosis and reduces the number of mature gametes in animal models [187]. Animal studies have shown that maternal cannabinoid exposure can induce subtle reproductive and endocrine changes in the offspring, including altered hormone profiles and gametogenesis efficiency [165,188]. Epigenetic analyses in exposed male rodents and human sperm demonstrate DNA methylation alterations in genes related to reproduction and neurodevelopment, suggesting potential intergenerational transmission [164,166].

Direct human evidence for transgenerational effects remains limited, but convergent animal and epigenetic data provide biological plausibility. Avoiding cannabis use during pregnancy therefore remains the most reliable strategy to protect both immediate fetal development and the germline health of future generations.

## 8. Knowledge Gaps and Future Directions

Major uncertainties remain about which cannabis constituents, which exposure patterns (dose, potency, route, smoked/vaped/edibles, frequency), and which gestational windows drive placental dysfunction and adverse fetal outcomes. Mechanistic studies often use supraphysiological concentrations and simplified models, while epidemiological studies are limited by self-report and polysubstance use (especially tobacco and alcohol), complicating causal inference.

Future research should therefore focus on the following: (1) better exposure measurement, combining validated questionnaires with objective biomarkers in maternal samples, cord blood/meconium, and placental tissue, plus details on product potency and route; (2) more realistic experimental models (placental explants, organoids, and trophoblast–endothelium systems) tested at clinically relevant mixtures and concentrations; (3) improved pharmacokinetic evidence, using ex vivo placenta perfusion and maternal–fetal PBPK models to define transfer over time, tissue accumulation, and transporter involvement, including in placental disease such as preeclampsia and fetal growth restriction; and (4) stronger epidemiological designs (e.g., sibling/within-family comparisons and negative controls) with better control for tobacco and mental health.

Finally, we need long-term follow-up of exposed children while carefully separating prenatal exposure from exposure via breastfeeding and clearer data on preconception exposure (including paternal use).

## 9. Conclusions

Current evidence supports abstaining from cannabis use during preconception, pregnancy, and lactation to optimize maternal, fetal, and intergenerational health outcomes. Nevertheless, cannabis use during pregnancy is on the rise.

This review has examined the effects of cannabis, particularly THC and CBD, on the placenta and the ECS. The placenta plays a vital role in nutrient exchange and fetal development, and its development, function, and blood flow can be influenced by the ECS. From this perspective, it is not surprising that THC and CBD use has deleterious effects during pregnancy, with potential long-term consequences for the developing infant.

Both for CBD and THC, there is a wide range in dose, frequency, and timing of exposure. It is unlikely that controlled studies with these drugs during pregnancy will ever be performed. This implies that further insights into their kinetics (e.g., placental passage), as well as those of other cannabinoids, rely on ex vivo models like the isolated perfused placenta. Mechanistic studies in placental organoids, placental explants, placental blood vessels, and trophoblast cell lines might help to unravel how cannabinoids affect placental development and blood flow. A detailed follow-up of the offspring of cannabis-using mothers is required to obtain hard outcome data on the potential long-term effects of cannabis use.

## Data Availability

No new data were created or analyzed in this study. Data sharing is not applicable to this article.

## References

[1] Chadi N., Levy S. (2019). What Every Pediatric Gynecologist Should Know About Marijuana Use in Adolescents. J. Pediatr. Adolesc. Gynecol..

[2] Namdar D., Anis O., Poulin P., Koltai H. (2020). Chronological Review and Rational and Future Prospects of Cannabis-Based Drug Development. Molecules.

[3] Koren G., Cohen R. (2020). The use of cannabis for Hyperemesis Gravidarum (HG). J. Cannabis Res..

[4] Thayyil B., Yusuf K. (2025). Evidence on the effect of in-utero cannabis exposure in neonates. J. Perinatol..

[5] Mendez-Reyes H.F., Franco-Olaya M., Canon-Cubillos O., Uribe-Lopez J.M., Delgado-Alvarez M.C., Velasquez-Portilla M. (2024). Olaya-CM Morphological and clinical findings in placentas and newborns with a history of tobacco, alcohol, and other substance abuse during pregnancy. J. Neonatal-Perinat. Med..

[6] Brown Q.L., Sarvet A.L., Shmulewitz D., Martins S.S., Wall M.M., Hasin D.S. (2017). Trends in Marijuana Use Among Pregnant and Nonpregnant Reproductive-Aged Women, 2002–2014. J. Am. Med. Assoc..

[7] Pratt Tremblay G., Dimanlig-Cruz S., Dion A., Corsi D.J. (2025). Trends in Prenatal Substance Use Across Ontario, Canada. JAMA Netw. Open.

[8] Singh S., Filion K., Abenhaim H., Eisenberg M. (2020). Prevalence and outcomes of prenatal recreational cannabis use in high-income countries: A scoping review. BJOG Int. J. Obstet. Gynaecol..

[9] Volkow N.D., Han B., Compton W.M., McCance-Katz E.F. (2019). Self-reported Medical and Nonmedical Cannabis Use Among Pregnant Women in the United States. J. Am. Med. Assoc..

[10] Agolli A., Agolli O., Chowdhury S., Shet V., Benitez J.S.C., Bheemisetty N., Waleed M.S. (2022). Increased cannabis use in pregnant women during COVID-19 pandemic. Discoveries.

[11] Denson R.K., Guerrero M., Bond W., Patterson A., May G., Polatsek T., Mermelstein R.J. (2025). Beliefs and perceived benefits and harms of perinatal cannabis use among pre- and post-pregnancy women. Drug Alcohol Depend. Rep..

[12] Radwan M.M., Chandra S., Gul S., Elsohly M.A. (2021). Cannabinoids, Phenolics, Terpenes and Alkaloids of Cannabis. Molecules.

[13] Elsohly M.A., Slade D. (2005). Chemical constituents of marijuana: The complex mixture of natural cannabinoids. Life Sci..

[14] Pourseyed Lazarjani M., Torres S., Hooker T., Fowlie C., Young O., Seyfoddin A. (2020). Methods for quantification of cannabinoids: A narrative review. J. Cannabis Res..

[15] Jang S.-Y., Jin H.-L., Yu G.-R., Lim D.-W., Park W.-H. (2025). Cannabis sativa Root Extract Exerts Anti-Nociceptive and Anti-Inflammatory Effects via Endocannabinoid Pathway Modulation In Vivo and In Vitro. Int. J. Mol. Sci..

[16] Gołyszny M., Dragon J., Obuchowicz E. (2025). Role of interplay between endocannabinoids and neuropeptides in pathogenesis and therapy of depressive and anxiety disorders. Neuropeptides.

[17] Pacher P., Kogan N.M., Mechoulam R. (2020). Beyond THC and Endocannabinoids. Annu. Rev. Pharmacol. Toxicol..

[18] Thakur G.A., Duclos R.I., Makriyannis A. (2005). Natural cannabinoids: Templates for drug discovery. Life Sci..

[19] Maia J., Fonseca B.M., Teixeira N., Correia-da-Silva G. (2020). The fundamental role of the endocannabinoid system in endometrium and placenta: Implications in pathophysiological aspects of uterine and pregnancy disorders. Hum. Reprod. Update.

[20] Harhangi M.S., Simons S.H.P., Bijma H.H., Nguyen A., Nguyen T.-V., Kaitu’u-Lino T., Reiss I.K., Danser A.H., Broekhuizen M. (2025). Placental Endocannabinoid System: Focus on Preeclampsia and Cannabis Use. Hypertension.

[21] Melchiorre K., Giorgione V., Thilaganathan B. (2022). The placenta and preeclampsia: Villain or victim?. Am. J. Obstet. Gynecol..

[22] Kumar A.R., Sheikh E.D., Monson J.W., Ligon S.E., Talley R.L., Dornisch E.M., Howitz K.J., Damicis J.R., Ieronimakis N., Unadkat J.D. (2023). Understanding the Mechanism and Extent of Transplacental Transfer of (−)-∆^9^-Tetrahydrocannabinol (THC) in the Perfused Human Placenta to Predict In Vivo Fetal THC Exposure. Clin. Pharmacol. Ther..

[23] Berman E., Erenburg N., Beloosesky R., Eyal S., Kovo M. (2023). Placental disposition of cannabidiol: An ex vivo perfusion study. Epilepsia.

[24] Matias I., Gonthier M., Petrosino S., Docimo L., Capasso R., Hoareau L., Monteleone P., Roche R., A Izzo A., Di Marzo V. (2007). Role and regulation of acylethanolamides in energy balance: Focus on adipocytes and *β*-cells. Br. J. Pharmacol..

[25] Taylor A.H., Bachkangi P., Konje J.C. (2023). Labour and premature delivery differentially affect the expression of the endocannabinoid system in the human placenta. Histochem. Cell Biol..

[26] Park B., Gibbons H.M., Mitchell M.D., Glass M. (2003). Identification of the CB1 Cannabinoid Receptor and Fatty Acid Amide Hydrolase (FAAH) in the Human Placenta. Placenta.

[27] Trabucco E., Acone G., Marenna A., Pierantoni R., Cacciola G., Chioccarelli T., Mackie K., Fasano S., Colacurci N., Meccariello R. (2009). Endocannabinoid system in first trimester placenta: Low FAAH and high CB1 expression characterize spontaneous miscarriage. Placenta.

[28] Wei B.Q., Mikkelsen T.S., McKinney M.K., Lander E.S., Cravatt B.F. (2006). A Second Fatty Acid Amide Hydrolase with Variable Distribution among Placental Mammals. J. Biol. Chem..

[29] Krzyżewska A., Baranowska-Kuczko M., Mińczuk K., Kozłowska H. (2021). Cannabinoids—A New Perspective in Adjuvant Therapy for Pulmonary Hypertension. Int. J. Mol. Sci..

[30] Greenhough A., Smartt H.J.M., Moore A.E., Roberts H.R., Williams A.C., Paraskeva C., Kaidi A. (2009). The COX-2/PGE2 pathway: Key roles in the hallmarks of cancer and adaptation to the tumour microenvironment. Carcinogenesis.

[31] Lee K., Hardy D.B. (2021). Metabolic Consequences of Gestational Cannabinoid Exposure. Int. J. Mol. Sci..

[32] Mathew B., Lakshminrusimha S. (2017). Persistent Pulmonary Hypertension in the Newborn. Children.

[33] Suchopár J., Laštůvka Z., Mašková S., Alblová M., Pařízek A. (2021). Endocannabinoids. Česká Gynekol..

[34] Feduniw S., Krupa I., Łagowska K., Laudański P., Tabarkiewicz J., Stawarz B., Raba G. (2024). Placental Cannabinoid Receptor Expression in Preterm Birth. J. Pregnancy.

[35] Rokeby A.C.E., Natale B.V., Natale D.R.C. (2023). Cannabinoids and the placenta: Receptors, signaling and outcomes. Placenta.

[36] Habayeb O.M.H., Taylor A.H., Bell S.C., Taylor D.J., Konje J.C. (2008). Expression of the Endocannabinoid System in Human First Trimester Placenta and Its Role in Trophoblast Proliferation. Endocrinology.

[37] Costa M.A., Fonseca B.M., Marques F., Teixeira N.A., Correia-Da-Silva G. (2015). The psychoactive compound of Cannabis sativa, Δ^9^-tetrahydrocannabinol (THC) inhibits the human trophoblast cell turnover. Toxicology.

[38] Chang X., Bian Y., He Q., Yao J., Zhu J., Wu J., Wang K., Duan T. (2017). Suppression of STAT3 Signaling by Δ^9^-Tetrahydrocannabinol (THC) Induces Trophoblast Dysfunction. Cell. Physiol. Biochem..

[39] Neradugomma N.K., Drafton K., Mor G.G., Mao Q. (2019). Marijuana-derived cannabinoids inhibit uterine endometrial stromal cell decidualization and compromise trophoblast-endometrium cross-talk. Reprod. Toxicol..

[40] Lojpur T., Easton Z., Raez-Villanueva S., Laviolette S., Holloway A.C., Hardy D.B. (2019). Δ^9^-Tetrahydrocannabinol leads to endoplasmic reticulum stress and mitochondrial dysfunction in human BeWo trophoblasts. Reprod. Toxicol..

[41] Blázquez C., Casanova M.L., Planas A., del Pulgar T.G., Villanueva C., Fernández-Aceñero M.J., Aragonés J., Huffman J.W., Jorcano J.L., Guzmán M. (2003). Inhibition of tumor angiogenesis by cannabinoids. FASEB J..

[42] Dobovišek L., Krstanović F., Borštnar S., Debeljak N. (2020). Cannabinoids and Hormone Receptor-Positive Breast Cancer Treatment. Cancers.

[43] Kita N., Mitsushita J., Ohira S., Takagi Y., Ashida T., Kanai M., Nikaido T., Konishi I. (2003). Expression and Activation of MAP Kinases, ERK1/2, in the Human Villous Trophoblasts. Placenta.

[44] Socała K., Jakubiec M., Abram M., Mlost J., Starowicz K., Kamiński R.M., Ciepiela K., Andres-Mach M., Zagaja M., Metcalf C.S. (2024). TRPV1 channel in the pathophysiology of epilepsy and its potential as a molecular target for the development of new antiseizure drug candidates. Prog. Neurobiol..

[45] Costa M.A., Fonseca B.M., Keating E., Teixeira N.A., Correia-Da-Silva G. (2014). Transient receptor potential vanilloid 1 is expressed in human cytotrophoblasts: Induction of cell apoptosis and impairment of syncytialization. Int. J. Biochem. Cell Biol..

[46] Contassot E., Wilmotte R., Tenan M., Belkouch M.-C., Schnüriger V., de Tribolet N., Bourkhardt K., Dietrich P.-Y. (2004). Arachidonylethanolamide Induces Apoptosis of Human Glioma Cells through Vanilloid Receptor-1. J. Neuropathol. Exp. Neurol..

[47] Zhang Y., Liang P., Yang L., Shan K.Z., Feng L., Chen Y., Liedtke W., Coyne C.B., Yang H. (2022). Functional coupling between TRPV4 channel and TMEM16F modulates human trophoblast fusion. eLife.

[48] De Clercq K., Pinto S., Van Den Broek E., Voets T., Vriens J. (2019). Placental TRPV2 expression is indispensable for normal fetal growth. Placenta.

[49] Allerkamp H.H., Bondarenko A.I., Tawfik I., Kamali-Simsek N., Horvat Mercnik M., Madreiter-Sokolowski C.T., Wadsack C. (2025). In vitro examination of Piezo1-TRPV4 dynamics: Implications for placental endothelial function in normal and preeclamptic pregnancies. Am. J. Physiol.-Cell Physiol..

[50] Janssens A., Silvestri C., Martella A., Vanoevelen J.M., Di Marzo V., Voets T. (2018). Δ^9^-tetrahydrocannabivarin impairs epithelial calcium transport through inhibition of TRPV5 and TRPV6. Pharmacol. Res..

[51] Ramírez-Orozco R.E., García-Ruiz R., Morales P., Villalón C.M., Villafán-Bernal J.R., Marichal-Cancino B.A. (2019). Potential metabolic and behavioural roles of the putative endocannabinoid receptors GPR18, GPR55 and GPR119 in feeding. Curr. Neuropharmacol..

[52] Yang H., Zhou J., Lehmann C. (2016). GPR55—A putative “type 3” cannabinoid receptor in inflammation. J. Basic Clin. Physiol. Pharmacol..

[53] Kremshofer J., Siwetz M., Berghold V.M., Lang I., Huppertz B., Gauster M. (2015). A role for GPR55 in human placental venous endothelial cells. Histochem. Cell Biol..

[54] Ryberg E., Larsson N., Sjögren S., Hjorth S., Hermansson N., Leonova J., Elebring T., Nilsson K., Drmota T., Greasley P.J. (2007). The orphan receptor GPR55 is a novel cannabinoid receptor. Br. J. Pharmacol..

[55] Lottero-Leconte R., Lara A., Plaza J., Arroyo-Salvo C., Bogetti M.E., Rivolta A.E.Y., Dellavalle F., Sengiali F., Cetica P., Rio S. (2025). Role of GPR55 receptor in bovine sperm capacitation. Andrology.

[56] Console-Bram L., Brailoiu E., Brailoiu G.C., Sharir H., Abood M.E. (2014). Activation of GPR18 by cannabinoid compounds: A tale of biased agonism. Br. J. Pharmacol..

[57] Ulu A., Sahoo P.K., Yuil-Valdes A.G., Mukherjee M., Van Ormer M., Muthuraj P.G., Thompson M., Berry A.A., Hanson C.K., Natarajan S.K. (2019). Omega-3 Fatty Acid-Derived Resolvin D2 Regulates Human Placental Vascular Smooth Muscle and Extravillous Trophoblast Activities. Int. J. Mol. Sci..

[58] Odori S., Hosoda K., Tomita T., Fujikura J., Kusakabe T., Kawaguchi Y., Doi R., Takaori K., Ebihara K., Sakai Y. (2013). GPR119 expression in normal human tissues and islet cell tumors: Evidence for its islet-gastrointestinal distribution, expression in pancreatic beta and alpha cells, and involvement in islet function. Metabolism.

[59] Lauffer L.M., Iakoubov R., Brubaker P.L. (2009). GPR119 Is Essential for Oleoylethanolamide-Induced Glucagon-Like Peptide-1 Secretion from the Intestinal Enteroendocrine L-Cell. Diabetes.

[60] Parast M.M., Yu H., Ciric A., Salata M.W., Davis V., Milstone D.S. (2009). PPARγ Regulates Trophoblast Proliferation and Promotes Labyrinthine Trilineage Differentiation. PLoS ONE.

[61] Daoud G., Simoneau L., Masse A., Rassart E., Lafond J. (2005). Expression of cFABP and PPAR in trophoblast cells: Effect of PPAR ligands on linoleic acid uptake and differentiation. Biochim. Biophys. Acta (BBA)—Mol. Cell Biol. Lipids.

[62] El Dairi R., Huuskonen P., Pasanen M., Rysä J. (2018). Peroxisome proliferator activated receptor gamma (PPAR-γ) ligand pioglitazone regulated gene networks in term human primary trophoblast cells. Reprod. Toxicol..

[63] Rios C., Gomes I., Devi L.A. (2006). *μ* opioid and CB1 cannabinoid receptor interactions: Reciprocal inhibition of receptor signaling and neuritogenesis. Br. J. Pharmacol..

[64] Mantione K.J., Angert R.M., Cadet P., Kream R.M., Stefano G.B. (2010). Identification of a µ opiate receptor signaling mechanism in human placenta. Med. Sci. Monit..

[65] Fonseca B.M., Correia-Da-Silva G., Almada M., Costa M.A., Teixeira N.A. (2013). The Endocannabinoid System in the Postimplantation Period: A Role during Decidualization and Placentation. Int. J. Endocrinol..

[66] Costa M.A. (2016). The endocannabinoid system: A novel player in human placentation. Reprod. Toxicol..

[67] Molvarec A., Fügedi G., Szabó E., Stenczer B., Walentin S., Rigó J. (2015). Decreased circulating anandamide levels in preeclampsia. Hypertens. Res..

[68] Lombó M., Giommi C., Paolucci M., Notarstefano V., Montik N., Carpini G.D., Ciavattini A., Ragusa A., Maradonna F., Giorgini E. (2022). Preeclampsia Correlates with an Increase in Cannabinoid Receptor 1 Levels Leading to Macromolecular Alterations in Chorionic Villi of Term Placenta. Int. J. Mol. Sci..

[69] Westerberg A.C., Degnes M.-H.L., Andresen I.J., Sellevoll H.B., Lekva T., Ueland T., Bergman L., Henriksen T., Roland M.C.P., Zucknick M. (2025). Placenta-Derived Proteins in Preeclampsia: A Human In Vivo Study. Hypertension.

[70] Shamran H., Singh N.P., Zumbrun E.E., Murphy A., Taub D.D., Mishra M.K., Price R.L., Chatterjee S., Nagarkatti M., Nagarkatti P.S. (2017). Fatty acid amide hydrolase (FAAH) blockade ameliorates experimental colitis by altering microRNA expression and suppressing inflammation. Brain Behav. Immun..

[71] Dong C., Chen J., Harrington A., Vinod K.Y., Hegde M.L., Hegde V.L. (2019). Cannabinoid exposure during pregnancy and its impact on immune function. Cell. Mol. Life Sci..

[72] Huppertz B. (2008). The anatomy of the normal placenta. J. Clin. Pathol..

[73] Braustein G.D., Buster J.E., Soares J.R., Gross S.J. (1983). Pregnancy hormone concentrations in marijuana users. Life Sci..

[74] Maia J., Almada M., Midão L., Fonseca B.M., Braga J., Gonçalves D., Teixeira N., Correia-Da-Silva G. (2020). The Cannabinoid Delta-9-tetrahydrocannabinol Disrupts Estrogen Signaling in Human Placenta. Toxicol. Sci..

[75] Almada M., Amaral C., Oliveira A., Fernandes P.A., Ramos M.J., Fonseca B.M., Correia-Da-Silva G., Teixeira N. (2020). Cannabidiol (CBD) but not tetrahydrocannabinol (THC) dysregulate in vitro decidualization of human endometrial stromal cells by disruption of estrogen signaling. Reprod. Toxicol..

[76] Rosario G.X., Brown S., Karmakar S., Rumi M.A.K., Nayak N.R. (2025). Super-Enhancers in Placental Development and Diseases. J. Dev. Biol..

[77] Khare M., Taylor A.H., Konje J.C., Bell S.C. (2006). Δ^9^-tetrahydrocannabinol inhibits cytotrophoblast cell proliferation and modulates gene transcription. Mol. Hum. Reprod..

[78] Walker O.S., Ragos R., Gurm H., Lapierre M., May L.L., Raha S. (2020). Delta-9-tetrahydrocannabinol disrupts mitochondrial function and attenuates syncytialization in human placental BeWo cells. Physiol. Rep..

[79] Walker O.S., Gurm H., Sharma R., Verma N., May L.L., Raha S. (2021). Delta-9-tetrahydrocannabinol inhibits invasion of HTR8/SVneo human extravillous trophoblast cells and negatively impacts mitochondrial function. Sci. Rep..

[80] Alves P., Amaral C., Teixeira N., Correia-da-Silva G. (2023). Effects of a combination of cannabidiol and delta-9-tetrahydrocannabinol on key biological functions of HTR-8/SVneo extravillous trophoblast cells. Toxicology.

[81] Shustorovich A., Corroon J., Wallace M.S., Sexton M. (2024). Biphasic effects of cannabis and cannabinoid therapy on pain severity, anxiety, and sleep disturbance: A scoping review. Pain Med..

[82] Koven J.L., Natale B.V., Hardy D.B., Natale D.R.C. (2025). Delta 9-Tetrahydrocannabinol Signaling Through Cannabinoid Receptor 1 Alters Trophoblast Differentiation. Stem Cells Dev..

[83] Alves P., Amaral C., Teixeira N., Correia-da-Silva G. (2021). Cannabidiol disrupts apoptosis, autophagy and invasion processes of placental trophoblasts. Arch. Toxicol..

[84] Podinic T., Limoges L., Monaco C., MacAndrew A., Minhas M., Nederveen J., Raha S. (2024). Cannabidiol Disrupts Mitochondrial Respiration and Metabolism and Dysregulates Trophoblast Cell Differentiation. Cells.

[85] Barnett G., Chiang C.W.N., Perez-Reyes M., Owens S.M. (1982). Kinetic study of smoking marijuana. J. Pharmacokinet. Biopharm..

[86] Adams I.B., Martin B.R. (1996). Cannabis: Pharmacology and toxicology in animals and humans. Addiction.

[87] Schwope D.M., Karschner E.L., Gorelick D.A., Huestis M.A. (2011). Identification of Recent Cannabis Use: Whole-Blood and Plasma Free and Glucuronidated Cannabinoid Pharmacokinetics following Controlled Smoked Cannabis Administration. Clin. Chem..

[88] Okada H., Tsuzuki T., Murata H. (2018). Decidualization of the human endometrium. Reprod. Med. Biol..

[89] Chang X., Li H., Li Y., He Q., Yao J., Duan T., Wang K. (2018). RhoA/MLC signaling pathway is involved in Δ^9^-tetrahydrocannabinol-impaired placental angiogenesis. Toxicol. Lett..

[90] Natale B.V., Gustin K.N., Lee K., Holloway A.C., Laviolette S.R., Natale D.R.C., Hardy D.B. (2020). Δ^9^-tetrahydrocannabinol exposure during rat pregnancy leads to symmetrical fetal growth restriction and labyrinth-specific vascular defects in the placenta. Sci. Rep..

[91] Allen S., Natale B.V., Ejeckam A.O., Lee K., Hardy D.B., Natale D.R.C. (2024). Cannabidiol Exposure During Rat Pregnancy Leads to Labyrinth-Specific Vascular Defects in the Placenta and Reduced Fetal Growth. Cannabis Cannabinoid Res..

[92] Solinas M., Massi P., Cantelmo A., Cattaneo M., Cammarota R., Bartolini D., Cinquina V., Valenti M., Vicentini L., Noonan D. (2012). Cannabidiol inhibits angiogenesis by multiple mechanisms. Br. J. Pharmacol..

[93] Domino E.F., Rennick P., Pearl J.H. (1974). Dose-effect relations of marijuana smoking on various physiological parameters in experienced male users. Observations on limits of self-titration of intake. Clin. Pharmacol. Ther..

[94] Cheung C.P., Coates A.M., Baker R.E., Burr J.F. (2024). Acute Effects of Cannabis Inhalation on Arterial Stiffness, Vascular Endothelial Function, and Cardiac Function. J. Am. Heart Assoc..

[95] Cheung C.P., Baker R.E., Coates A.M., Burr J.F. (2025). The Acute Cardiovascular Response to Dynamic Exercise and Recovery Following Cannabis Use. Am. J. Physiol. Heart Circ. Physiol..

[96] Brik M., Sandonis M., Gil J., Hernandez-Fleury A., Parramón-Puig G., Maiz N., Suy A., Carreras E. (2022). Intrauterine cannabis exposure and fetal and maternal blood flow: A case-control study. Acta Obs. Gynecol. Scand..

[97] El Marroun H., Tiemeier H., Steegers E.A., Roos-Hesselink J.W., Jaddoe V.W., Hofman A., Verhulst F.C., Brink W.v.D., Huizink A.C. (2010). A prospective study on intrauterine cannabis exposure and fetal blood flow. Early Hum. Dev..

[98] Brar B.K., Patil P.S., Jackson D.N., Gardner M.O., Alexander J.M., Doyle N.M. (2021). Effect of intrauterine marijuana exposure on fetal growth patterns and placental vascular resistance. J. Matern.-Fetal Neonatal Med..

[99] Roberts V.H.J., Schabel M.C., Boniface E.R., D’mEllo R.J., Morgan T.K., Terrobias J.J.D., Graham J.A., Borgelt L.M., Grant K.A., Sullivan E.L. (2022). Chronic prenatal delta-9-tetrahydrocannabinol exposure adversely impacts placental function and development in a rhesus macaque model. Sci. Rep..

[100] Ellis E.F., Moore S.F., Willoughby K.A. (1995). Anandamide and delta 9-THC dilation of cerebral arterioles is blocked by indomethacin. Am. J. Physiol..

[101] Fleming I., Schermer B., Popp R., Busse R. (1999). Inhibition of the production of endothelium-derived hyperpolarizing factor by cannabinoid receptor agonists. Br. J. Pharmacol..

[102] Zygmunt P.M., Andersson D.A., Hogestatt E.D. (2002). Delta 9-tetrahydrocannabinol and cannabinol activate capsaicin-sensitive sensory nerves via a CB1 and CB2 cannabinoid receptor-independent mechanism. J. Neurosci..

[103] O’Sullivan S.E., Kendall D.A., Randall M.D. (2005). The effects of Delta9-tetrahydrocannabinol in rat mesenteric vasculature, and its interactions with the endocannabinoid anandamide. Br. J. Pharmacol..

[104] O’Sullivan S.E., Tarling E.J., Bennett A.J., Kendall D.A., Randall M.D. (2005). Novel time-dependent vascular actions of Delta9-tetrahydrocannabinol mediated by peroxisome proliferator-activated receptor gamma. Biochem. Biophys. Res. Commun..

[105] O’Sullivan S.E., Kendall D.A., Randall M.D. (2006). Further characterization of the time-dependent vascular effects of delta9-tetrahydrocannabinol. J. Pharmacol. Exp. Ther..

[106] O’Sullivan S.E., Randall M.D., Gardiner S.M. (2007). The In Vitro and In Vivo Cardiovascular Effects of Δ^9^-Tetrahydrocannabinol in Rats Made Hypertensive by Chronic Inhibition of Nitric-Oxide Synthase. J. Pharmacol. Exp. Ther..

[107] O’Sullivan S.E., Sun Y., Bennett A.J., Randall M.D., Kendall D.A. (2009). Time-dependent vascular actions of cannabidiol in the rat aorta. Eur. J. Pharmacol..

[108] Stanley C.P., Hind W.H., Tufarelli C., O’Sullivan S.E. (2015). Cannabidiol causes endothelium-dependent vasorelaxation of human mesenteric arteries via CB1 activation. Cardiovasc. Res..

[109] Baranowska-Kuczko M., Kozłowska H., Kloza M., Sadowska O., Kozłowski M., Kusaczuk M., Kasacka I., Malinowska B. (2020). Vasodilatory effects of cannabidiol in human pulmonary and rat small mesenteric arteries: Modification by hypertension and the potential pharmacological opportunities. J. Hypertens..

[110] Harbison R.D., Mantilla-Plata B. (1972). Prenatal toxicity, maternal distribution and placental transfer of tetrahydrocannabinol. J. Pharmacol. Exp. Ther..

[111] Martin B.R., Dewey W.L., Harris L.S., Beckner J.S. (1977). 3H-delta9-tetrahydrocannabinol distribution in pregnant dogs and their fetuses. Res. Commun. Chem. Pathol. Pharmacol..

[112] Bailey J.R., Cunny H.C., Paule M.G., Slikker W. (1987). Fetal disposition of Δ^9^-tetrahydrocannabinol (THC) during late pregnancy in the rhesus monkey. Toxicol. Appl. Pharmacol..

[113] Blackard C., Tennes K. (1984). Human placental transfer of cannabinoids. N. Engl. J. Med..

[114] Kumar A.R., Benson L.S., Wymore E.M., Phipers J.E., Dempsey J.C., Cort L.A., Unadkat J.D. (2025). Quantification and prediction of human fetal (-)-Δ^9^-tetrahydrocannabinol/(±)-11-OH-Δ^9^-tetrahydrocannabinol exposure during pregnancy to inform fetal cannabis toxicity. Nat. Commun..

[115] Chen X., Gáborik Z., Mao Q., Unadkat J.D. (2024). Are Δ^9^-Tetrahydrocannabinol and Its Major Metabolites Substrates or Inhibitors of Placental or Human Hepatic Drug Solute-Carrier Transporters?. Int. J. Mol. Sci..

[116] Chen X., Unadkat J.D., Mao Q. (2021). Tetrahydrocannabinol and Its Major Metabolites Are Not (or Are Poor) Substrates or Inhibitors of Human P-Glycoprotein [ATP-Binding Cassette (ABC) B1] and Breast Cancer Resistance Protein (ABCG2). Drug Metab. Dispos..

[117] Fisher S.E., Atkinson M., Chang B. (1987). Effect of delta-9-tetrahydrocannabinol on the in vitro uptake of alpha-amino isobutyric acid by term human placental slices. Pediatr. Res..

[118] Feinshtein V., Erez O., Ben-Zvi Z., Erez N., Eshkoli T., Sheizaf B., Sheiner E., Huleihel M., Holcberg G. (2013). Cannabidiol changes P-gp and BCRP expression in trophoblast cell lines. PeerJ.

[119] Feinshtein V., Erez O., Ben-Zvi Z., Eshkoli T., Sheizaf B., Sheiner E., Holcberg G. (2013). Cannabidiol enhances xenobiotic permeability through the human placental barrier by direct inhibition of breast cancer resistance protein: An ex vivo study. Am. J. Obstet. Gynecol..

[120] Portillo R., Abad C., Synova T., Kastner P., Heblik D., Kucera R., Karahoda R., Staud F. (2024). Cannabidiol disrupts tryptophan metabolism in the human term placenta. Toxicology.

[121] van Zundert S.K., Broekhuizen M., Smit A.J., van Rossem L., Mirzaian M., Willemsen S.P., Danser A.H., De Rijke Y.B., Reiss I.K., Merkus D. (2022). The Role of the Kynurenine Pathway in the (Patho) physiology of Maternal Pregnancy and Fetal Outcomes: A Systematic Review. Int. J. Tryptophan Res..

[122] Broekhuizen M., Klein T., Hitzerd E., De Rijke Y.B., Schoenmakers S., Sedlmayr P., Danser A.H., Merkus D., Reiss I.K. (2020). ʟ-Tryptophan–Induced Vasodilation Is Enhanced in Preeclampsia. Hypertension.

[123] Oláh A., Markovics A., Szabó-Papp J., Szabó P.T., Stott C., Zouboulis C.C., Bíró T. (2016). Differential effectiveness of selected non-psychotropic phytocannabinoids on human sebocyte functions implicates their introduction in dry/seborrhoeic skin and acne treatment. Exp. Dermatol..

[124] Authement A.K., Isoherranen N. (2024). The impact of pregnancy and associated hormones on the pharmacokinetics of Δ^9^-tetrahydrocannabinol. Expert Opin. Drug Metab. Toxicol..

[125] Monfort A., Ferreira E., Leclair G., Lodygensky G.A. (2022). Pharmacokinetics of Cannabis and Its Derivatives in Animals and Humans During Pregnancy and Breastfeeding. Front. Pharmacol..

[126] Huestis M.A. (2007). Human Cannabinoid Pharmacokinetics. Chem. Biodivers..

[127] Lucas C.J., Galettis P., Schneider J. (2018). The pharmacokinetics and the pharmacodynamics of cannabinoids. Br. J. Clin. Pharmacol..

[128] Newmeyer M.N., Swortwood M.J., Barnes A.J., Abulseoud O.A., Scheidweiler K.B., Huestis M.A. (2016). Free and Glucuronide Whole Blood Cannabinoids’ Pharmacokinetics after Controlled Smoked, Vaporized, and Oral Cannabis Administration in Frequent and Occasional Cannabis Users: Identification of Recent Cannabis Intake. Clin. Chem..

[129] Grotenhermen F. (2003). Pharmacokinetics and Pharmacodynamics of Cannabinoids. Clin. Pharmacokinet..

[130] Poyatos L., Pérez-Acevedo A.P., Papaseit E., Pérez-Mañá C., Martin S., Hladun O., Siles A., Torrens M., Busardo F.P., Farré M. (2020). Oral Administration of Cannabis and Δ-9-tetrahydrocannabinol (THC) Preparations: A Systematic Review. Medicina.

[131] Ohlsson A., Lindgren J.E., Wahlen A., Agurell S., Hollister L.E., Gillespie H.K. (1980). Plasma delta-9-tetrahydrocannabinol concentrations and clinical effects after oral and intravenous administration and smoking. Clin. Pharmacol. Ther..

[132] Grant K.S., Petroff R., Isoherranen N., Stella N., Burbacher T.M. (2018). Cannabis use during pregnancy: Pharmacokinetics and effects on child development. Pharmacol. Ther..

[133] Ahmed A.I.A., Van Den Elsen G.A.H., Colbers A., Kramers C., Burger D.M., Van Der Marck M.A., Olde Rikkert M.G.M. (2015). Safety, pharmacodynamics, and pharmacokinetics of multiple oral doses of delta-9-tetrahydrocannabinol in older persons with dementia. Psychopharmacology.

[134] Wall M.E., Sadler B.M., Brine D., Taylor H., Perez-Reyes M. (1983). Metabolism, disposition, and kinetics of delta-9-tetrahydrocannabinol in men and women. Clin. Pharmacol. Ther..

[135] Hunt C.A., Jones R.T. (1980). Tolerance and disposition of tetrahydrocannabinol in man. J. Pharmacol. Exp. Ther..

[136] Ward R.M., Varner M.W. (2019). Principles of Pharmacokinetics in the Pregnant Woman and Fetus. Clin. Perinatol..

[137] Ujváry I., Hanuš L. (2016). Human Metabolites of Cannabidiol: A Review on Their Formation, Biological Activity, and Relevance in Therapy. Cannabis Cannabinoid Res..

[138] Isoherranen N., Thummel K.E. (2013). Drug Metabolism and Transport During Pregnancy: How Does Drug Disposition Change during Pregnancy and What Are the Mechanisms that Cause Such Changes?. Drug Metab. Dispos..

[139] Yamaori S., Ebisawa J., Okushima Y., Yamamoto I., Watanabe K. (2011). Potent inhibition of human cytochrome P450 3A isoforms by cannabidiol: Role of phenolic hydroxyl groups in the resorcinol moiety. Life Sci..

[140] Davison J.M., Dunlop W., Ezimokhai M. (1980). 24-h Creatinine Clearance during the Third Trimester of Normal Pregnancy. BJOG Int. J. Obstet. Gynaecol..

[141] Patilea-Vrana G.I., Unadkat J.D. (2021). Development and Verification of a Linked Δ^9^-THC/11-OH-THC Physiologically Based Pharmacokinetic Model in Healthy, Nonpregnant Population and Extrapolation to Pregnant Women. Drug Metab. Dispos..

[142] Kouthouridis S., Sotra A., Khan Z., Alvarado J., Raha S., Zhang B. (2023). Modeling the Progression of Placental Transport from Early- to Late-Stage Pregnancy by Tuning Trophoblast Differentiation and Vascularization. Adv. Healthc. Mater..

[143] Włoch S., Pałasz A., Kamiński M. (2009). Active and passive transport of drugs in the human placenta. Ginekol. Pol..

[144] Collier A.C. (2002). Metabolizing enzyme localization and activities in the first trimester human placenta: The effect of maternal and gestational age, smoking and alcohol consumption. Hum. Reprod..

[145] Hakkola J., Pelkonen O., Pasanen M., Raunio H. (1998). Xenobiotic-Metabolizing Cytochrome P450 Enzymes in the Human Feto-Placental Unit: Role in Intrauterine Toxicity. Crit. Rev. Toxicol..

[146] Abrams R.M., Cook C.E., Davis K.H., Niederreither K., Jaeger M.J., Szeto H.H. (1985). Plasma delta-9-tetrahydrocannabinol in pregnant sheep and fetus after inhalation of smoke from a marijuana cigarette. Alcohol. Drug Res..

[147] Hutchings D.E., Martin B.R., Gamagaris Z., Miller N., Fico T. (1989). Plasma concentrations of delta-9-tetrahydrocannabinol in dams and fetuses following acute or multiple prenatal dosing in rats. Life Sci..

[148] Ryan S.A., Ammerman S.D., O’Connor M.E., Gonzalez L., Patrick S.W., Quigley J., Walker L.R., Meek J.Y., Johnston M., Stellwagen L. (2018). Marijuana Use During Pregnancy and Breastfeeding: Implications for Neonatal and Childhood Outcomes. Pediatrics.

[149] Kumar A.R., Patilea-Vrana G.I., Anoshchenko O., Unadkat J.D. (2022). Characterizing and Quantifying Extrahepatic Metabolism of (−)-Δ^9^-Tetrahydrocannabinol (THC) and Its Psychoactive Metabolite, (±)-11-Hydroxy-Δ^9^-THC (11-OH-THC). Drug Metab. Dispos..

[150] Pang X.-Y., Cheng J., Kim J.-H., Matsubara T., Krausz K.W., Gonzalez F.J. (2012). Expression and Regulation of Human Fetal-Specific CYP3A7 in Mice. Endocrinology.

[151] Ekström L., Johansson M., Rane A. (2013). Tissue Distribution and Relative Gene Expression of UDP-Glucuronosyltransferases (2B7, 2B15, 2B17) in the Human Fetus. Drug Metab. Dispos..

[152] Lacroix D., Sonnier M., Moncion A., Cheron G., Cresteil T. (1997). Expression of CYP3A in the Human Liver—Evidence that the Shift between CYP3A7 and CYP3A4 Occurs Immediately After Birth. Eur. J. Biochem..

[153] Moscovitz J., Aleksunes L. (2013). Establishment of Metabolism and Transport Pathways in the Rodent and Human Fetal Liver. Int. J. Mol. Sci..

[154] Green V.R., Kennedy-Hendricks A., Saloner B., Bandara S. (2024). Substance use and treatment characteristics among pregnant and non-pregnant females, 2015–2019. Drug Alcohol Depend..

[155] el Marroun H., Tiemeier H., Jaddoe V.W.V., Hofman A., Mackenbach J.P., Steegers E.A.P., Verhulst F.C., van den Brink W., Huizink A.C. (2008). Demographic, emotional and social determinants of cannabis use in early pregnancy: The Generation R study. Drug Alcohol Depend..

[156] Crume T.L., Juhl A.L., Brooks-Russell A., Hall K.E., Wymore E., Borgelt L.M. (2018). Cannabis Use During the Perinatal Period in a State with Legalized Recreational and Medical Marijuana: The Association Between Maternal Characteristics, Breastfeeding Patterns, and Neonatal Outcomes. J. Pediatr..

[157] Young-Wolff K.C., Adams S.R., Alexeeff S.E., Zhu Y., Chojolan E., Slama N.E., Zhu Y., Chojolan E., Slama N.E., Does M.B. (2024). Prenatal Cannabis Use and Maternal Pregnancy Outcomes. JAMA Intern. Med..

[158] Weisbeck S.J., Bright K.S., Ginn C.S., Smith J.M., Hayden K.A., Ringham C. (2021). Perceptions about cannabis use during pregnancy: A rapid best-framework qualitative synthesis. Can. J. Public Health.

[159] Badowski S., Smith G. (2020). Cannabis use during pregnancy and postpartum. Can. Fam. Physician.

[160] Joseph M.D., Krivorotko D., Koenig M.R., Wesselink A.K., Eisenberg M.L., Sommer G.J., Rothman K.J., Stuver S.O., Hatch E.E., Wise L.A. (2025). A North American preconception cohort study of cannabis use and semen quality. Andrology.

[161] Payne K.S., Mazur D.J., Hotaling J.M., Pastuszak A.W. (2019). Cannabis and Male Fertility: A Systematic Review. J. Urol..

[162] Pizzol D., Demurtas J., Stubbs B., Soysal P., Mason C., Isik A.T., Solmi M., Smith L., Veronese N. (2019). Relationship Between Cannabis Use and Erectile Dysfunction: A Systematic Review and Meta-Analysis. Am. J. Men’s Health.

[163] Meah F., Lundholm M., Emanuele N., Amjed H., Poku C., Agrawal L., Emanuele M.A. (2022). The effects of cannabis and cannabinoids on the endocrine system. Rev. Endocr. Metab. Disord..

[164] Murphy S.K., Itchon-Ramos N., Visco Z., Huang Z., Grenier C., Schrott R., Acharya K., Boudreau M.H., Price T.M., Raburn D.J. (2018). Cannabinoid exposure and altered DNA methylation in rat and human sperm. Epigenetics.

[165] Meccariello R., Battista N., Bradshaw H.B., Wang H. (2014). Updates in Reproduction Coming from the Endocannabinoid System. Int. J. Endocrinol..

[166] Schrott R., Murphy S.K. (2020). Cannabis use and the sperm epigenome: A budding concern?. Environ. Epigenetics.

[167] Harlow A.F., Wesselink A.K., Hatch E.E., Rothman K.J., Wise L.A. (2021). Male Preconception Marijuana Use and Spontaneous Abortion. Epidemiology.

[168] Sood S., Trasande L., Mehta-Lee S.S., Brubaker S.G., Ghassabian A., Jacobson M.H. (2022). Maternal Cannabis Use in the Perinatal Period: Data from the Pregnancy Risk Assessment Monitoring System Marijuana Supplement, 2016–2018. J. Addict. Med..

[169] Duval C., Wyse B.A., Fuchs Weizman N., Kuznyetsova I., Madjunkova S., Librach C.L. (2025). Cannabis impacts female fertility as evidenced by an in vitro investigation and a case-control study. Nat. Commun..

[170] Fonseca B.M., Rebelo I. (2022). Cannabis and Cannabinoids in Reproduction and Fertility: Where We Stand. Reprod. Sci..

[171] Gabrhelík R., Mahic M., Lund I.O., Bramness J., Selmer R., Skovlund E., Handal M., Skurtveit S. (2021). Cannabis Use during Pregnancy and Risk of Adverse Birth Outcomes: A Longitudinal Cohort Study. Eur. Addict. Res..

[172] Lo J.O., Hedges J.C., Girardi G. (2022). Impact of cannabinoids on pregnancy, reproductive health, and offspring outcomes. Am. J. Obstet. Gynecol..

[173] Avalos L.A., Shenkute M., Alexeeff S.E., Oberman N., Croen L.A., Davignon M., Adams S.R., Ansley D., Castellanos C., Young-Wolff K.C. (2024). Maternal Prenatal Cannabis Use and Child Autism Spectrum Disorder. JAMA Netw. Open.

[174] Noble A.J., Adams A.T., Satsangi J., Boden J.M., Osborne A.J. (2025). Prenatal cannabis exposure is associated with alterations in offspring DNA methylation at genes involved in neurodevelopment, across the life course. Mol. Psychiatry.

[175] Gunn J.K.L., Rosales C.B., Center K.E., Nuñez A., Gibson S.J., Christ C., Ehiri J.E. (2016). Prenatal exposure to cannabis and maternal and child health outcomes: A systematic review and meta-analysis. BMJ Open.

[176] Bertrand K.A., Hanan N.J., Honerkamp-Smith G., Best B.M., Chambers C.D. (2018). Marijuana Use by Breastfeeding Mothers and Cannabinoid Concentrations in Breast Milk. Pediatrics.

[177] Baker T., Datta P., Rewers-Felkins K., Thompson H., Kallem R.R., Hale T.W. (2018). Transfer of Inhaled Cannabis into Human Breast Milk. Obstet. Gynecol..

[178] Wymore E.M., Palmer C., Wang G.S., Metz T.D., Bourne D.W.A., Sempio C., Bunik M. (2021). Persistence of Δ-9-Tetrahydrocannabinol in Human Breast Milk. JAMA Pediatr..

[179] Astley S.J., Little R.E. (1990). Maternal marijuana use during lactation and infant development at one year. Neurotoxicol. Teratol..

[180] Fernández-Ruiz J., Berrendero F., Hernández M.L., Ramos J.A. (2000). The endogenous cannabinoid system and brain development. Trends Neurosci..

[181] De Fonseca F.R., Ramos J.A., Bonnin A., Fernández-Ruiz J.J. (1993). Presence of cannabinoid binding sites in the brain from early postnatal ages. NeuroReport.

[182] Hines L.A., Spry E.A., Moreno-Betancur M., Husin H.M., Becker D., Middleton M., Craig J.M., Doyle L.W., Olsson C.A., Patton G. (2021). Cannabis and tobacco use prior to pregnancy and subsequent offspring birth outcomes: A 20-year intergenerational prospective cohort study. Sci. Rep..

[183] Leeder J.S., Gaedigk R., Marcucci K.A., Gaedigk A., Vyhlidal C.A., Schindel B.P., Pearce R.E. (2005). Variability of CYP3A7 Expression in Human Fetal Liver. J. Pharmacol. Exp. Ther..

[184] Campolongo P., Trezza V., Palmery M., Trabace L., Cuomo V. (2009). Chapter 9 Developmental Exposure to Cannabinoids Causes Subtle and Enduring Neurofunctional Alterations.

[185] Cobellis G., Cacciola G., Scarpa D., Meccariello R., Chianese R., Franzoni M.F., Mackie K., Pierantoni R., Fasano S. (2006). Endocannabinoid System in Frog and Rodent Testis: Type-1 Cannabinoid Receptor and Fatty Acid Amide Hydrolase Activity in Male Germ Cells1. Biol. Reprod..

[186] Grimaldi P., Orlando P., Di Siena S., Lolicato F., Petrosino S., Bisogno T., Geremia R., Petrocellis L., Marzo V. (2009). The endocannabinoid system and pivotal role of the CB_2_ receptor in mouse spermatogenesis. Proc. Natl. Acad. Sci. USA.

[187] De Domenico E., Todaro F., Rossi G., Dolci S., Geremia R., Rossi P., Grimaldi P. (2017). Overactive type 2 cannabinoid receptor induces meiosis in fetal gonads and impairs ovarian reserve. Cell Death Dis..

[188] Dalterio S., Badr F., Bartke A., Mayfield D. (1982). Cannabinoids in Male Mice: Effects on Fertility and Spermatogenesis. Science.

